# The Complexity of the Relationship Between Mitral and Aortic Valve Annular Dimensions in the Same Healthy Adults: Detailed Insights from the Three-Dimensional Speckle-Tracking Echocardiographic MAGYAR-Healthy Study

**DOI:** 10.3390/biomedicines14020304

**Published:** 2026-01-29

**Authors:** Attila Nemes, Barbara Bordács, Nóra Ambrus, Csaba Lengyel

**Affiliations:** Department of Medicine, Albert Szent-Györgyi Medical School, University of Szeged, 6725 Szeged, Hungary; bordacs.barbara.aniko@med.u-szeged.hu (B.B.); ambrusnora@gmail.com (N.A.); lengyel.csaba@med.u-szeged.hu (C.L.)

**Keywords:** three-dimensional, speckle-tracking, echocardiography, mitral, aortic valve, annulus, healthy

## Abstract

**Introduction.** Although the aortic valve and mitral valve differ significantly in structure, function, and location, they both play a significant role in left ventricular (LV) function. The aim of the current study was to analyze the relationship between the mitral valve annulus (MVA) and the aortic valve annulus (AVA), as measured by three-dimensional speckle-tracking echocardiography (3DSTE) in the same healthy individuals with average or smaller/larger annular diameters (Ds), areas (As), and perimeters (Ps) in end-diastole (D) and end-systole (S). **Methods.** This study comprised 134 healthy adult participants with a mean age of 31.0 (16.0) years (73 males). A complete medical investigation included physical examination, laboratory tests, standard 12-lead electrocardiography, and two-dimensional Doppler echocardiography supplemented with 3DSTE. **Results.** Almost all end-diastolic and end-systolic MVA dimensions increased significantly with enlarging MVA. Similarly, as MVA-D-D and MVA-P-D increased, nearly all end-diastolic and end-systolic AVA dimensions exhibited a positive trend. Lower-than-average MVA-A-D was associated with a trend toward higher AVA dimensions (excluding AVA-P-D) compared to the mean MVA-A-D; conversely, higher-than-average MVA-A-D was also associated with increased AVA dimensions. AVA perimeter values were notably higher than those recorded in the lower-than-average MVA-A-D subgroup. In subjects with lower-than-average end-diastolic MVA dimensions, a non-significantly higher proportion of larger end-systolic AVA was observed relative to end-diastolic AVA. While AVA dimensions remained unchanged despite increasing MVA-D-S, a positive trend in AVA dimensions—reaching statistical significance for certain parameters—was observed alongside increasing MVA-A-S and MVA-P-S. In subjects with lower-than-average end-systolic MVA dimensions, there was a non-significantly higher prevalence of larger end-systolic AVA compared to end-diastolic AVA. Furthermore, nearly all end-diastolic and end-systolic AVA dimensions increased significantly with increasing AVA. Increases in AVA-D-D, AVA-A-D, and AVA-P-D were generally accompanied by a trend toward higher end-diastolic and end-systolic MVA dimensions; however, MVA-D-S peaked in the presence of lower-than-average end-diastolic AVA dimensions. In subjects with lower-than-average end-diastolic AVA, a non-significantly higher proportion of larger end-systolic AVA was noted compared to end-diastolic AVA. Notably higher MVA parameters were observed in the presence of mean AVA-D-S and AVA-A-S compared to their lower-than-average counterparts. Finally, end-diastolic MVA parameters showed a positive trend with increasing AVA-P-S, and subjects with higher-than-average end-systolic AVA dimensions demonstrated a significantly higher proportion of larger end-systolic AVA compared to end-diastolic AVA. **Conclusions.** There is a strong and complex association between the dimensions of the MVA and AVA, as assessed by 3DSTE, when measured simultaneously in the same healthy adults.

## 1. Introduction

Although the mitral (MV) and aortic valves (AV) have differences in location, structure, and function, they play a significant role in left ventricular (LV) function [1,2,3,4,5,6]. The MV and its annulus (MVA) are the inlet of the LV, while the AV and its annulus (AVA) are its outlet [1,2,3,4,5,6]. Modern imaging techniques can help to assess the annuli of both valves, thus helping to understand their functional cooperation during the cardiac cycle, as has already been demonstrated for the MVA and tricuspid valve annulus [7]. One such method is three-dimensional speckle-tracking echocardiography (3DSTE), which is suitable for the non-invasive characterization of MVA and AVA in addition to chamber quantifications, even in the same case, simultaneously [7,8,9,10,11,12,13]. Although this has not yet been confirmed in detail with cardiovascular imaging studies in healthy individuals, it can be hypothesized that there is an MVA-AVA coupling. This means that there is a relationship between the size of the annulus of the two valves and their changes during the cardiac cycle, since they belong to the same heart chamber, the LV [1,2,3,4,5,6]. It is also hypothesized that changes in the annulus of one valve affect the size of the other, both in diastole and in systole. Therefore, the aim of the current study was to analyze the relationship between MVA and AVA by simultaneously measuring their dimensions using 3DSTE in the same healthy individuals. What happens when MVA/AVA is average-sized or smaller/larger was also examined. To the best of the authors’ knowledge, this has never been investigated before in healthy subjects using a non-invasive cardiovascular imaging technique like 3DSTE.

## 2. Subjects and Methods

**Subject population**. This study comprised 134 healthy adult participants with a mean age of 34.7 ± 12.3 years (73 males), who joined the study voluntarily between 2011 and 2017. Complete medical investigation included physical examination, laboratory tests, standard 12-lead electrocardiography (ECG), and two-dimensional (2D) Doppler echocardiography supplemented with 3DSTE. All parameters proved to be within normal reference ranges. None of the individuals had a positive medical history with any known disorder or pathology (self-reported), or were regular medication takers (self-reported), smokers (self-reported), obese (body mass index > 30 kg/m^2^), pregnant (self-reported), or athletes (no regular sport training in the last 2 years and/or not a registered member of a sport club). This study was part of the ‘**M**otion **A**nalysis of the heart and **G**reat vessels b**Y** three-dimension**A**l speckle-t**R**acking echocardiography in **Healthy** subjects’ **(MAGYAR-Healthy) Study**, which was conducted with the aim of performing physiological analyses between 3DSTE-derived parameters, among others (‘Magyar’ means ‘Hungarian’ in the Hungarian language). This study was conducted in accordance with the Declaration of Helsinki (as revised in 2013). The Institutional and Regional Human Biomedical Research Committee of the University of Szeged, Hungary, approved the study under registration number 71/2011, with the latest approval on 17 March 2025. All study participants gave written informed consent.

**Blood Pressure Assessment.** Following the collection of demographic and clinical baseline characteristics, systolic and diastolic blood pressures (SBP and DBP, respectively) were measured using a mercury-column sphygmomanometer on the left arm. Measurements were performed in the supine position after a 10 min rest period. SBP and DBP were identified based on the first and fifth Korotkoff sounds, respectively. Participants were required to abstain from stimulants for at least 30 min prior to the procedure. The reported values represent the arithmetic mean of three consecutive measurements [12,13].

**Two-dimensional Doppler echocardiography.** In all cases, the same Toshiba Artida^®^ cardiac ultrasound tool attached to a 1–5 MHz broadband PST-30BT phased-array probe (Toshiba Medical Systems, Tokyo, Japan) was used. The routine 2D Doppler echo analysis included left atrial (LA) and LV chamber quantifications with modified Simpson’s measurement of LV ejection fraction (EF). To exclude significant valvular regurgitation and stenosis on any valves, Doppler echocardiography was used together with determination of early (E) and late (A) diastolic velocities of transmitral inflow and their ratio (E/A) [14].

**Three-dimensional speckle-tracking echocardiography.** The 3DSTE analysis was carried out in 2 steps [7,8,9,10,11,12,13]. As a first step, using the same Toshiba Artida^TM^ echocardiographic tool, 3D echocardiographic data acquisitions were performed from the apical window following a probe change to a 3D-capable one, namely the PST-25SX matrix-array transducer (Toshiba Medical Systems, Tokyo, Japan). For optimal images, settings were optimized, and subjects were in a breath-hold state. A total of 6 subvolumes were acquired within 6 cardiac cycles, and datasets were stored digitally for later analysis.

**Figure 1:**
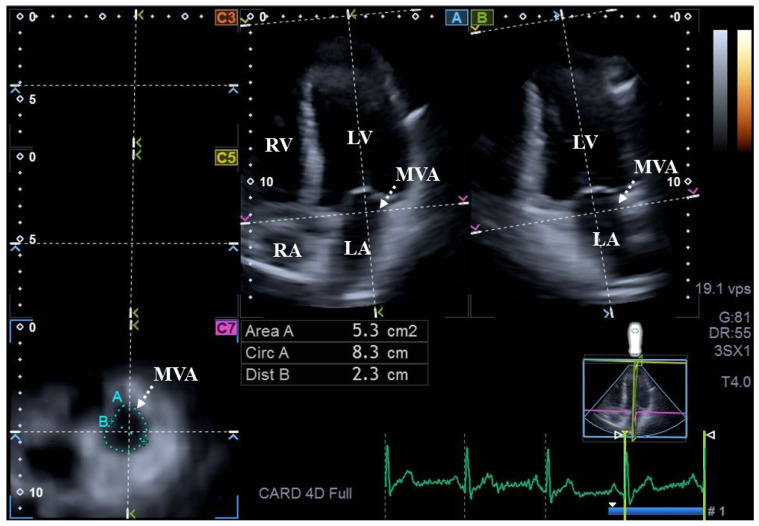
Three-dimensional speckle-tracking echocardiography-derived assessment of mitral annular dimensions in a healthy adult in end-diastole: (A) apical four-chamber and (B) apical two-chamber long-axis views and cross-sectional view (C7) of the mitral annulus optimized on A and B images. Mitral annular plane is indicated by white dotted arrows. **Abbreviations.** Area = mitral valve annular area, Circ = mitral valve annular perimeter, Dist = mitral valve annular diameter, LA = left atrium, LV = left ventricle, RA = right atrium, and RV = right ventricle.

**Figure 2:**
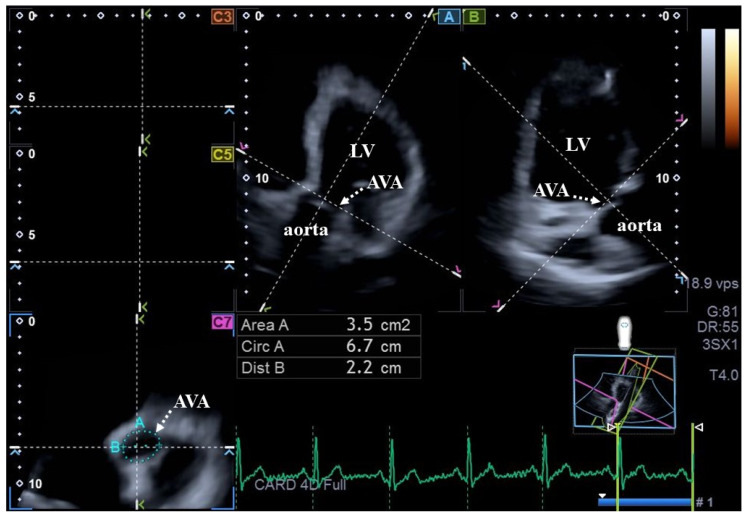
Three-dimensional speckle-tracking echocardiography-derived assessment of aortic valve annular dimensions in a healthy adult in end-diastole: (A) apical four-chamber and (B) apical two-chamber long-axis views and cross-sectional view (C7) of the aortic valve annulus optimized on (A,B) images. Aortic valve annular plane is indicated by white arrows. **Abbreviations:** Area = aortic valve annular area, Circ = aortic valve annular perimeter, Dist = maximum aortic valve annular diameter, LV = left ventricle, RV = right ventricle.

As the second step, during offline data analysis, a vendor-derived software named 3D Wall Motion Tracking (version 2.7, UltraExtend, Toshiba Medical Systems, Tokyo, Japan) was utilized. End-diastole and end-systole were defined with respect to electrical activity at the peak R wave and at the end of the T wave, respectively. For measurement of MVA dimensions, following definitions of the septal and lateral endpoints of the MVA on apical two- (AP2CH) and four-chamber (AP4CH) long-axis views, the following MVA dimensions were measured at end-diastole (D) and at end-systole (S) on C7 short-axis view (Figure 1): MVA diameter (MVA-D), MVA area (MVA-A), and MVA perimeter (MVA-P), all measured during planimetry. For measurement of AVA dimensions, optimal LV longitudinal planes were determined using AP2CH and AP4CH long-axis views. After visualization of the aortic valve/aorta by tilting and optimizing the longitudinal planes in long-axis views, the planes were positioned parallel to the central aortic root midline. The C7 cross-sectional view, to which the AVA was aligned, was positioned perpendicular to the longitudinal plane. Special attention had to be paid to ensuring that C7 was truly perpendicular to the longitudinal plane and that assessments were not taken in the sinus of Valsalva or in the LV outflow tract. Similar to MVA, the following AVA dimensions were measured at both end-diastole and end-systole (Figure 2): AVA diameter (AVA-D), AVA area (AVA-A), and AVA perimeter (AVA-P). All measurements were carried out using planimetric images [12,13].

**Statistical analysis**. Continuous variables were assessed for normality using the Shapiro–Wilk test. Normally distributed variables were expressed as mean ± standard deviation (SD), whereas non-normally distributed variables were presented as median and interquartile range (IQR). For normally distributed variables, analysis of variance (ANOVA) and independent samples t-tests were used for comparisons among three and two groups, respectively, with post hoc pairwise comparisons adjusted using the Bonferroni correction to account for multiple testing. For non-normally distributed variables, the Kruskal–Wallis test was applied for three-group comparisons, followed, when appropriate, by Dunn’s post hoc test, while two-group comparisons were performed using the Mann–Whitney U test. Categorical data were expressed in percentage (%) format. Fischer’s exact test was used for all categorical variables. The Bland–Altman method was used for intraobserver and interobserver agreements. For intraobserver and interobserver correlations, intraclass correlation coefficients (ICCs) were measured. Pearson’s correlation coefficients were calculated for correlations. Measurements were performed on 35 randomly selected subjects. Statistical significance was defined as p less than 0.05. All statistical analyses were carried out using SPSS version 29.0.0.0. (SPSS Inc., Chicago, IL, USA).

## 3. Results

**Clinical and 2D Doppler echocardiographic data.** All data were within normal reference ranges and are presented in Table 1. None of the subjects showed equal to or larger than grade 1 valvular regurgitation or early signs of valvular stenosis on any valves.

**Classification of subjects.** Mean ± SD of 3DSTE-derived MVA and AVA diameters, areas, and perimeters measured in end-diastole and end-systole are presented in Table 2. Based on these data, healthy subjects were classified into three subgroups. The following cut-offs were used (Table 3, Table 4, Table 5 and Table 6):

For MVA-D-D: 2.01 cm and 2.86 cm, while for MVA-D-S: 1.30 cm and 1.99 cm.

For MVA-A-D: 5.20 cm^2^ and 9.58 cm^2^, while for MVA-A-S: 2.27 cm^2^ and 4.67 cm^2^.

For MVA-P-D: 8.77 cm and 11.75 cm, while for MVA-P-S: 5.93 cm and 8.29 cm.

For AVA-D-D: 1.66 cm and 2.34 cm, while for AVA-D-S: 1.71 cm and 2.35 cm.

For AVA-A-D: 2.23 cm^2^ and 4.03 cm^2^, while for AVA-A-S: 2.41 cm^2^ and 4.19 cm^2^.

For AVA-P-D: 5.30 cm and 7.18 cm, while for AVA-P-S: 5.56 cm and 7.34 cm.

**Table 2:** Three-dimensional speckle-tracking echocardiography-derived mitral and tricuspid annular dimensions and functional properties.

Parameters	Measures
end-diastolic mitral valve annular diameter (MVA-D-D, cm)	2.45 ± 0.41 *
end-diastolic mitral valve annular area (MVA-A-D, cm^2^)	7.39 ± 2.19 *
end-diastolic mitral valve annular perimeter (MVA-P-D, cm)	10.26 ± 1.49 *
end-systolic mitral valve annular diameter (MVA-D-S, cm)	1.61 ± 0.38 *
end-systolic mitral valve annular area (MVA-A-S, cm^2^)	3.47 ± 1.20
end-systolic mitral valve annular perimeter (MVA-P-S, cm)	7.11 ± 1.18 *
end-diastolic aortic valve annular diameter (AVA-D-D, cm)	2.00 ± 0.34
end-diastolic aortic valve annular area (AVA-A-D, cm^2^)	3.13 ± 0.90
end-diastolic aortic valve annular perimeter (AVA-P-D, cm)	6.24 ± 0.94
end-systolic aortic valve annular diameter (AVA-D-S, cm)	2.03 ± 0.32
end-systolic aortic valve annular area (AVA-A-S, cm^2^)	3.30 ± 0.89
end-systolic aortic valve annular perimeter (AVA-P-S, cm)	6.45 ± 0.89

* *p* < 0.05 vs. AVA counterpart.

**Table 3:** Mitral valve and aortic valve annular parameters in different end-diastolic mitral valve annular groups.

	MVA-D-D ≤ 2.01 cm (n = 24)	2.01 cm < MVA-D-D < 2.86 cm (n = 88)	2.86 cm ≤ MVA-D-D (n = 22)	MVA-A-D ≤ 5.20 cm^2^ (n = 21)	5.20 cm^2^ < MVA-A-D < 9.58 cm^2^ (n = 77)	9.58 cm^2^ ≤ MVA-A-D (n = 36)	MVA-P-D ≤ 8.77 cm (n = 23)	8.77 cm < MVA-P-D < 11.75 cm (n = 89)	11.75 cm ≤ MVA-P-D (n = 22)
MVA-D-S (cm)	1.44 ± 0.29 *	1.63 ± 0.41 *,†	1.71 ± 0.30 *,†	1.43 ± 0.24 *	1.61 ± 0.39 *,†	1.72 ± 0.38 *,†	1.46 ± 0.23 *	1.59 ± 0.39 *	1.78 ± 0.37 *,†,‡
MVA-A-S (cm^2^)	2.85 ± 0.79	3.50 ± 1.24 †	4.08 ± 1.02 †,‡	2.54 ± 0.66 *	3.46 ± 1.13 †	4.04 ± 1.24 †,‡	2.53 ± 0.62 *	3.47 ± 1.15 *,†	4.36 ± 1.09 *,†,‡
MVA-P-S (cm)	6.64 ± 1.06	7.06 ± 1.17 *	7.86 ± 0.93 *,†,‡	6.10 ± 0.92	7.12 ± 1.04 *,†	7.69 ± 1.18 *,†,‡	6.06 ± 0.79	7.12 ± 1.08 *,†	8.04 ± 0.99 *,†,‡
MVA-D-D (cm)	1.90 ± 0.15	2.05 ± 0.35 *,†	3.09 ± 0.19 *,†,‡	2.00 ± 0.22	2.39 ± 0.33 *,†	2.84 ± 0.29 *,†,‡	2.03 ± 0.20	2.43 ± 0.35 *,†	2.91 ± 0.30 *,†,‡
MVA-A-D (cm^2^)	5.09 ±0.98 *	7.35 ± 1.66 *,†	10.16 ± 1.81 *,†,‡	4.40 ± 0.61 *	6.92 ± 0.88 *,†	10.28 ± 1.34 *,†,‡	4.47 ± 0.66 *	7.22 ± 1.18 *,†	10.88 ± 1.25 *,†,‡
MVA-P-D (cm)	8.84 ± 1.13 *	10.28 ± 1.21 *,†	11.86 ± 1.14 *,†,‡	8.07 ± 0.67 *	10.02 ± 0.59 *,†	12.17 ± 0.80 *,†,‡	8.05 ± 0.58 *	10.23 ± 0.74 *,†	12.55 ± 0.70 *,†,‡
AVA-D-S (cm)	1.98 ± 0.26	2.03 ± 0.32	2.10 ± 0.34	2.11 ± 0.31	1.98 ± 0.31	2.10 ± 0.32 ‡	2.09 ± 0.34	1.99 ± 0.30	2.12 ± 0.31
AVA-A-S (cm^2^)	3.12 ± 0.76	3.30 ± 0.90	3.56 ± 0.93	3.42 ± 0.85	3.15 ± 0.88	3.58 ± 0.89 ‡	3.39 ± 0.94	3.19 ± 0.86	3.61 ± 0.87 ‡
AVA-P-S (cm)	6.27 ± 0.76	6.46 ± 0.90	6.70 ± 0.93	6.56 ± 0.77	6.30 ± 0.89	6.75 ± 0.88 †,‡	6.50 ± 0.90	6.35 ± 0.86	6.79 ± 0.86 ‡
AVA-D-D (cm)	1.90 ± 0.29	2.02 ± 0.33	2.05 ± 0.35	1.97 ± 0.30	1.95 ± 0.34	2.12 ± 0.31 ‡	1.98 ± 0.31	1.97 ± 0.33	2.13 ± 0.33 ‡
AVA-A-D (cm^2^)	2.88 ± 0.98	3.17 ± 0.87	3.33 ± 0.88	3.17 ± 1.03	3.00 ± 0.86	3.37 ± 0.82 ‡	3.18 ± 1.02	3.07 ± 0.90	3.41 ± 0.89
AVA-P-D (cm)	5.77 ± 1.07	6.34 ± 0.86 †	6.49 ± 0.91 †	6.01 ± 1.18	6.17 ± 0.88	6.53 ± 0.81 †,‡	6.04 ± 1.17	6.16 ± 0.95	6.58 ± 0.87 ‡
ES-AVA-A > ED-AVA-A (%)	17 (71)	51 (58)	13 (59)	16 (76)	42 (55)	23 (64)	16 (70)	50 (56)	15 (63)

**Abbreviations.** MVA-D = mitral valve annular diameter, MVA-A = mitral valve annular area, MVA-P = mitral valve annular perimeter, AVA-D = aortic valve annular diameter, AVA-A = aortic valve annular area, AVA-P = aortic valve annular perimeter, S = end-systolic, and D = end-diastolic; * *p* < 0.05 vs. AVA counterpart; † *p* < 0.05 vs. mean − SD MVA-D dimension; ‡ *p* < 0.05 vs. mean MVA-D dimension; ES-AVA-A > ED-AVA-A = end-systolic AVA-A is larger than end-diastolic AVA-A.

**Table 4:** Mitral valve and aortic valve annular parameters in different end-systolic mitral valve annular groups.

	MVA-D-S ≤ 1.3 cm (n = 25)	1.3 cm < MVA-D-S < 1.99 cm (n = 83)	1.99 cm ≤ MVA-D-S (n = 26)	MVA-A-S ≤ 2.27 cm^2^ (n = 22)	2.27 cm^2^ < MVA-A-S < 4.67 cm^2^ (n = 90)	4.67 cm^2^ ≤ MVA-A-S (n = 22)	MVA-P-S ≤ 5.93 cm (n = 25)	5.93 cm < MVA-P-S < 8.29 cm (n = 87)	8.29 cm ≤ MVA-P-S (n = 22)
MVA-D-S (cm)	1.11 ± 0.11 *	1.58 ± 0.19 *,†	2.21 ± 0.17 *,†,‡	1.21 ± 0.14 *	1.58 ± 0.31 *,†	2.12 ± 0.25 †,‡	1.22 ± 0.15 *	1.64 ± 0.33 *,†	1.91 ± 0.41 †,‡
MVA-A-S (cm^2^)	2.32 ± 0.67 *	3.36 ± 0.88 *,†	5.03 ± 0.94 *,†,‡	1.90 ± 0.18 *	3.37 ± 0.66 †	5.47 ± 0.68 *,†,‡	2.04 ± 0.44 *	3.45 ± 0.73 †	5.14 ± 1.08 *,†,‡
MVA-P-S (cm)	6.13 ± 1.06	7.05 ± 0.96 *,†	8.32 ± 0.88 *,†,‡	5.39 ± 0.34 *	7.11 ± 0.71 *,†	8.80 ± 0.68 *,†,‡	5.55 ± 0.63 *	7.14 ± 0.66 *,†	8.72 ± 0.87 *,†,‡
MVA-D-D (cm)	2.25 ± 0.38 *	2.43 ± 0.41 *	2.72 ± 0.28 *,†,‡	2.23 ± 0.29 *	2.44 ± 0.43 *,†	2.69 ± 0.29 *,†,‡	2.21 ± 0.27 *	2.46 ± 0.42 *,†	2.64 ± 0.37 *,†
MVA-A-D (cm^2^)	6.64 ± 1.93 *	7.14 ± 2.13 *	8.98 ± 1.89 *,†,‡	6.07 ± 1.76 *	7.29 ± 2.10 *,†	9.10 ± 1.83 *,†,‡	6.04 ± 1.63 *	7.33 ± 2.05 *,†	9.10 ± 2.03 *,†,‡
MVA-P-D (cm)	9.84 ± 1.35 *	10.12 ± 1.48 *	11.17 ± 1.33 *,†,‡	9.40 ± 1.33 *	10.20 ± 1.43 *,†	11.39 ± 1.18 *,†,‡	9.39 ± 1.26 *	10.20 ± 1.40 *,†	11.50 ± 1.21 *,†,‡
AVA-D-S (cm)	2.03 ± 0.35	2.04 ± 0.31	1.99 ± 0.31	1.97 ± 0.36	2.04 ± 0.31	2.04 ± 0.29	1.99 ± 0.33	2.01 ± 0.31	2.10 ± 0.31
AVA-A-S (cm^2^)	3.29 ± 1.06	3.31 ± 0.87	3.27 ± 0.80	3.22 ± 1.03	3.29 ± 0.89	3.40 ± 0.72	3.20 ± 0.95	3.26 ± 0.89	3.46 ± 0.86 ‡
AVA-P-S (cm)	6.44 ± 1.03	6.47 ± 0.86	6.42 ± 0.87	6.36 ± 0.99	6.44 ± 0.89	6.60 ± 0.76	6.35 ± 0.92	6.40 ± 0.89	6.67 ± 0.85 ‡
AVA-D-D (cm)	2.03 ± 0.36	1.99 ± 0.32	1.98 ± 0.37	1.99 ± 0.32	1.97 ± 0.35	2.13 ± 0.28 ‡	2.01 ± 0.30	1.95 ± 0.34	2.13 ± 0.33 ‡
AVA-A-D (cm^2^)	3.04 ± 1.09	3.17 ± 0.81	3.06 ± 0.97	2.93 ± 1.01	3.11 ± 0.87	3.40 ± 0.84	2.96 ± 0.95	3.08 ± 0.86	3.41 ± 0.93
AVA-P-D (cm)	6.20 ± 1.07	6.27 ± 0.87	6.18 ± 1.03	6.09 ± 1.00	6.20 ± 0.94	6.57 ± 0.82	6.14 ± 0.94	6.16 ± 0.93	6.58 ± 0.91 ‡
ES-AVA > ED-AVA (%)	18 (72)	47 (57)	16 (62)	18 (82)	51 (57)	12 (55)	19 (76)	48 (55)	14 (64)

**Abbreviations.** MVA-D = mitral valve annular diameter, MVA-A = mitral valve annular area, MVA-P = mitral valve annular perimeter, AVA-D = aortic valve annular diameter, AVA-A = aortic valve annular area, AVA-P = aortic valve annular perimeter, S = end-systolic, and D = end-diastolic; * *p* < 0.05 vs. AVA counterpart; † *p* < 0.05 vs. mean − SD MVA dimension; ‡ *p* < 0.05 vs. mean MVA dimension; ES-AVA-A > ED-AVA-A = end-systolic AVA-A is larger than end-diastolic AVA-A.

**Table 5:** Mitral valve and aortic valve annular parameters in different end-diastolic aortic valve annular groups.

	AVA-D-D ≤ 1.66 cm (n = 17)	1.66 cm < AVA-D-D < 2.34 (n = 89)	2.34 cm ≤ AVA-D-D (n = 28)	AVA-A-D ≤ 2.23 cm^2^ (n = 18)	2.23 cm^2^ < AVA-A-D < 4.03 cm^2^ (n = 100)	4.03 cm^2^ ≤ AVA-A-D (n = 16)	AVA-P-D ≤ 5.3 cm (n = 17)	5.3 cm < AVA-P-D < 7.18 cm (n = 100)	7.18 cm ≤ AVA-P-D (n = 17)
MVA-D-S (cm)	1.77 ± 0.41	1.58 ± 0.36 *,†	1.60 ± 0.39 *	1.71 ± 0.48	1.60 ± 0.36 *	1.46 ± 0.33 *	1.74 ± 0.48	1.60 ± 0.36 *	1.54 ± 0.38 *
MVA-A-S (cm^2^)	3.42 ± 0.96 *	3.41 ± 1.16	3.69 ± 1.37	3.32 ± 1.19 *	3.46 ± 1.15	3.58 ± 1.50 *	3.34 ± 1.15 *	3.45 ± 1.16	3.73 ± 1.45 *
MVA-P-S (cm)	6.72 ± 1.03 *	7.06 ± 1.13 *	7.34 ± 1.38	6.78 ± 1.17 *	7.11 ± 1.10 *	7.32 ± 1.53	6.76 ± 1.11 *	7.11 ± 1.12 *	7.42 ± 1.47
MVA-D-D (cm)	2.31 ± 0.41 *	2.44 ± 0.41 *	2.55 ± 0.39	2.41 ± 0.44 *	2.44 ± 0.39 *	2.49 ± 0.44	2.38 ± 0.48 *	2.44 ± 0.39 *	2.54 ± 0.41
MVA-A-D (cm^2^)	7.42 ± 0.99 *	7.38 ± 2.11 *	7.88 ± 2.52 *	7.07 ± 2.33 *	7.32 ± 2.01 *	7.88 ± 2.75 *	7.14 ± 2.59 *	7.32 ± 2.02 *	8.05 ± 2.59 *
MVA-P-D (cm)	6.96 ± 0.96 *	10.27 ± 1.42 *	10.58 ± 1.71 *	9.95 ± 1.52 *	10.23 ± 1.39 *	10.64 ± 1.86 *	9.99 ± 1.66 *	10.24 ± 1.39 *	10.72 ± 1.76 *
AVA-D-S (cm)	1.68 ± 0.24	2.01 ± 0.26 †	2.31 ± 0.27 †,‡	1.67 ± 0.20	2.03 ± 0.27 †	2.41 ± 0.24 †,‡	1.74 ± 0.25	2.02 ± 0.28 †	2.37 ± 0.26 †,‡
AVA-A-S (cm^2^)	2.39 ± 0.58	3.20 ± 0.72 †	4.18 ± 0.84 †,‡	2.26 ± 0.37	3.26 ± 0.71 †	4.60 ± 0.76 †,‡	2.34 ± 0.45	3.25 ± 0.71 †	4.55 ± 0.77 †,‡
AVA-P-S (cm)	5.51 ± 0.66	6.37 ± 0.73 †	7.32 ± 0.72 †,‡	5.37 ± 0.48	6.44 ± 0.70 †	7.73 ± 0.59 †	5.44 ± 0.57	6.42 ± 0.71 †	7.68 ± 0.61 †,‡
AVA-D-D (cm)	1.44 ± 0.16	1.96 ± 0.16 †	2.47 ± 0.16 †,‡	1.54 ± 0.21	2.00 ± 0.25 †	2.47 ± 0.25 †,‡	1.55 ± 0.21	1.99 ± 0.24 †	2.49 ± 0.23 †,‡
AVA-A-D (cm^2^)	1.95 ± 0.44	3.03 ± 0.66 †	4.17 ± 0.66 †,‡	1.82 ± 0.29	3.10 ± 0.51 †	7.43 ± 1.39 †,‡	2.01 ± 1.03	3.07 ± 0.51 †	4.62 ± 0.54 †,‡
AVA-P-D (cm)	4.99 ± 0.57	6.13 ± 0.70 †	7.34 ± 0.55 †,‡	4.89 ± 0.49	6.29 ± 0.53 †	12.14 ± 0.54 †,‡	4.61 ± 0.69	6.27 ± 0.51 †	7.72 ± 0.42 †,‡
ES-AVA > ED-AVA (%)	13 (76)	55 (62)	13 (46)	15 (83)	57 (57)	9 (56)	13 (76)	59 (59)	9 (53)

**Abbreviations.** MVA-D = mitral valve annular diameter, MVA-A = mitral valve annular area, MVA-P = mitral valve annular perimeter, AVA-D = aortic valve annular diameter, AVA-A = aortic valve annular area, AVA-P = aortic valve annular perimeter, S = end-systolic, and D = end-diastolic; * *p* < 0.05 vs. AVA counterpart; † *p* < 0.05 vs. mean − SD MVA dimension; ‡ *p* < 0.05 vs. mean MVA dimension; ES-AVA-A > ED-AVA-A = end-systolic AVA-A is larger than end-diastolic AVA-A.

**Table 6:** Mitral and aortic valve annular parameters in different end-systolic aortic valve annular groups.

	AVA-D-S ≤ 1.71 cm (n = 25)	1.71 cm < AVA-D-S < 2.35 cm (n = 89)	2.35 cm ≤ AVA-D-S (n = 20)	AVA-A-S ≤ 2.41 cm^2^ (n = 18)	2.41 cm^2^ < AVA-A-S < 4.19 cm^2^ (n = 92)	4.19 cm^2^ ≤ AVA-A-S (n = 24)	AVA-P-S ≤ 5.56 cm (n = 19)	5.56 cm < AVA-P-S < 7.34 cm (n = 93)	7.34 cm ≤ AVA-P-S (n = 22)
MVA-D-S (cm)	1.59 ± 0.34	1.63 ± 0.41 *	1.55 ± 0.29 *	1.59 ± 0.34	1.63 ± 0.40 *	1.52 ± 0.32 *	1.63 ± 0.37 *	1.63 ± 0.39 *	1.49 ± 0.33 *
MVA-A-S (cm^2^)	3.11 ± 1.07 *	3.60 ± 1.18 *	3.34 ± 1.35 *	3.14 ± 1.00 *	3.56 ± 1.22 *	3.39 ± 1.23 *	3.24 ± 1.06 *	3.52 ± 1.21 *	3.45 ± 1.28 *
MVA-P-S (cm)	6.67 ± 1.05 *	7.25 ± 1.13 *	7.02 ± 1.40	6.71 ± 1.02 *	7.19 ± 1.17 *	7.10 ± 1.26 *	6.78 ± 1.05 *	7.16 ± 1.16 *	7.18 ± 1.33
MVA-D-D (cm)	2.44 ± 0.37 *	2.44 ± 0.40 *	2.48 ± 0.47	2.47 ± 0.40 *	2.42 ± 0.40 *	2.53 ± 0.45	2.51 ± 0.41 *	2.40 ± 0.39 *	2.60 ± 0.43 *
MVA-A-D (cm^2^)	7.16 ± 1.72 *	7.55 ± 2.11 *	6.94 ± 2.86 *	6.96 ± 1.42 *	7.47 ± 2.17 *	7.40 ± 2.66 *	7.17 ± 1.65 *	7.36 ± 2.18 *	7.83 ± 2.54 *
MVA-P-D (cm)	10.18 ± 1.24 *	10.37 ± 1.44 *,†	9.92 ± 1.91 *	9.90 ± 1.07 *	10.34 ± 1.47 *	10.27 ± 1.79 *	10.02 ± 1.15 *	10.26 ± 1.51 *	10.61 ± 1.59 *
AVA-D-S (cm)	1.54 ± 0.13	2.05 ± 0.14 †	2.53 ± 0.13 †,‡	1.58 ± 0.20	2.01 ± 0.20 †	2.45 ± 0.20 †,‡	1.58 ± 0.20	2.02 ± 0.20 †	2.46 ± 0.19 †
AVA-A-S (cm^2^)	2.26 ± 0.50	3.29 ± 0.56 †	4.65 ± 0.69 †,‡	1.94 ± 0.26	3.20 ± 0.45 †	4.70 ± 0.50 †,‡	1.99 ± 0.32	3.23 ± 0.48 †	4.71 ± 0.52 †
AVA-P-S (cm)	5.31 ± 0.59	6.50 ± 0.56 †	7.66 ± 0.62 †,‡	5.00 ± 0.38	6.40 ± 0.47 †	7.54 ± 0.44 †,‡	5.02 ± 0.38	9.10 ± 0.55 †	7.79 ± 0.42 †
AVA-D-D (cm)	1.74 ± 0.30	2.00 ± 0.96 †	2.32 ± 0.31 †,‡	1.64 ± 0.24	1.98 ± 0.28 †	2.33 ± 0.29 †,‡	1.65 ± 0.24	6.43 ± 0.47 †	2.36 ± 0.27 †,‡
AVA-A-D (cm^2^)	2.27 ± 0.54	3.17 ± 0.77 †	4.00 ± 0.88 †,‡	2.06 ± 0.45	3.08 ± 0.72 †	4.10 ± 0.76 †,‡	2.06 ± 0.44	3.09 ± 0.71 †	4.15 ± 0.70 †,‡
AVA-P-D (cm)	5.43 ± 0.67	6.27 ± 0.85 †	7.11 ± 0.79 †,‡	5.16 ± 0.61	6.20 ± 0.79 †	7.22 ± 0.68 †,‡	5.16 ± 0.60	6.21 ± 0.78 †	7.27 ± 0.63 †,‡
ES-AVA > ED-AVA (%)	15 (60)	47 (53)	19 (95) †,‡	7 (39)	53 (58)	21 (88) †,‡	8 (42)	53 (57)	20 (91) †,‡

**Abbreviations.** MVA-D = mitral valve annular diameter, MVA-A = mitral valve annular area, MVA-P = mitral valve annular perimeter, AVA-D = aortic valve annular diameter, AVA-A = aortic valve annular area, AVA-P = aortic valve annular perimeter, S = end-systolic, and D = end-diastolic; * *p* < 0.05 vs. AVA counterpart; † *p* < 0.05 vs. mean − SD MVA dimension; ‡ *p* < 0.05 vs. mean MVA dimension; ES-AVA-A > ED-AVA-A = end-systolic AVA-A is larger than end-diastolic AVA-A.

**MVA versus AVA parameters.** End-diastolic MVA parameters were larger than their AVA counterparts. Some end-systolic MVA parameters were lower, whereas some of them were larger than their AVA counterparts (Table 2, Table 3, Table 4, Table 5 and Table 6).

**End-diastolic MVA dimensions and AVA.** Almost all end-diastolic and end-systolic MVA dimensions significantly increased with increasing end-diastolic MVA dimensions. With increasing MVA-D-D and MVA-P-D, almost all end-diastolic and end-systolic AVA dimensions showed a tendential increase. The lower-than-average MVA-A-D was associated with tendentiously higher AVA dimensions (except AVA-P-D) as compared to the average MVA-A-D. The higher-than-average MVA-A-D was associated with higher AVA dimensions as well. AVA perimeter data proved to be higher than those measured in the subgroup that had lower-than-average MVA-A-D. In subjects with lower-than-average end-diastolic MVA dimensions, a (non-significantly) higher proportion of larger end-systolic AVA was present compared with larger end-diastolic AVA (Table 3).

**End-systolic MVA dimensions and AVA.** Almost all end-diastolic and end-systolic MVA dimensions significantly increased with increasing end-systolic MVA dimensions. With increasing MVA-D-S, AVA dimensions proved to be the same. With increasing MVA-A-S and MVA-P-S, a tendentious increase in AVA dimensions could be detected, which proved to be significant among certain parameters. In subjects with lower-than-average end-systolic MVA dimensions, a (non-significantly) higher proportion of larger end-systolic AVA was present than larger end-diastolic AVA (Table 4).

**End-diastolic AVA dimensions and MVA.** Almost all end-diastolic and end-systolic AVA dimensions significantly increased with increasing end-diastolic AVA dimensions. With increasing AVA-D-D, AVA-A-D, and AVA-P-D, almost all end-diastolic and end-systolic MVA dimensions showed a tendentious increase, but MVA-D-S proved to be the highest in the presence of lower-than-mean end-diastolic AVA dimensions. In subjects with lower-than-average end-diastolic AVA dimensions, a (non-significantly) higher proportion of larger end-systolic AVA was present than larger end-diastolic AVA (Table 5).

**End-systolic AVA dimensions and MVA.** Almost all end-diastolic and end-systolic AVA dimensions significantly increased with increasing end-systolic AVA dimensions. Tendentiously higher MVA parameters could be detected in the presence of mean AVA-D-S and AVA-A-S as compared to lower-than-average counterparts. With increasing AVA-P-S, MVA-A-D and MVA-P-D showed a tendentious increase. In subjects with higher-than-average end-systolic AVA dimensions, a significantly higher proportion of larger end-systolic AVA was present than larger end-diastolic AVA (Table 6).

**Correlation analysis.** The correlation coefficients (r) with corresponding *p* values are presented in Table 7 and Table 8.

**Reproducibility of 3DSTE-derived MVA/AVA measurements.** Intraobserver and interobserver agreements of end-diastolic and end-systolic MVA and AVA diameters, areas, and perimeters are presented with their respective ICCs in Table 9.

## 4. Discussion

The MV and AV are the inlet and outlet of the same heart chamber, nominally the LV [1,2,3,4,5,6]. MV and AV differ not only in the number of leaflets (two vs. three), but their shape, tissue quality, and structure are also different. Moreover, papillary muscles and tendineal chords also have significant roles in the operation of the MV. At the beginning of systole, the MV is closed and the AV is open, while at the beginning of diastole, the valves are in opposite states. The annuli of these valves (MVA and AVA) have important, but different, roles during the cardiac cycle. In short, although both are made up of fibrotic tissue and are closely related to the surrounding tissue, primarily the LV muscle bands, MVA is also associated with the LA, while AVA is associated with the aorta [15]. According to these facts, while in end-diastole, the MVA is larger compared to its end-systolic counterpart; in the case of AVA, the size corresponding to the cardiac cycle is not so clear [7]. Regarding data from the literature, in approximately 60% of individuals, end-systolic AVA areas are greater, while in approximately 30%, the end-diastolic one is larger [13,16]. Since the LV pumping function, under healthy conditions, transports blood from the LA to the aorta in accordance with the cardiac cycle, the question may rightly arise as to what relationships can be demonstrated between the sizes of the MVA and AVA if they are of average size or larger/smaller [15].

The enormous progress that cardiovascular imaging has undergone can also be seen in our daily routine, as, for example, in the field of echocardiography, where many procedures now help our work. Although 3D echocardiography has been around for 20 years, it can still offer new possibilities [7,12,13]. Along with LV chamber quantification, using the same acquired 3D echocardiographic datasets, MVA and AVA can be assessed simultaneously, allowing physiological analysis, such as in this research [7,8,9,10,11,12,13]. In this case, the ability of 3D echocardiography to visualize MVA and AVA ‘en-face’ during a single examination using the same 3D database can be utilized [12,13,17,18]. Both 3D echocardiographic analysis of MVA and AVA are demonstrated in the literature, and 3DSTE-derived normal reference values with age- and gender-dependency for both annuli are published [12,13,17,18].

The present study has several findings. As expected, it was confirmed that in both MVA and AVA, an increase in any parameter characterizing end-diastolic or end-systolic size is accompanied by an increase in the other parameters of the same annulus, regardless of the phase of the cardiac cycle during which they were measured. It could be demonstrated that regardless of whether the MVA or AVA size was larger and in which phase of the cardiac cycle it was measured, an (tendential) increase in the annular dimensions of the other valve could be detected with significant correlations. However, this did not prove to be a general rule; in two cases, the findings were not so clear. First, most AVA dimensions were higher in the case of lower-than-mean MVA-A-D/MVA-A-P as compared to those measured in cases with mean MVA-A-D/MVA-A-P. Second, most MVA parameters were the highest in the presence of mean end-systolic AVA dimensions as compared to lower/higher-than-mean values. In addition to the above, interestingly, the proportion of cases with larger end-systolic AVA-A was higher in the presence of lower-than-mean MVA and AVA. These findings indicate the complexity of the relationship between the annular sizes of the two valves. One may rightly wonder what the reason for the above associations might be. The different structure of the valves and their annuli, their deformed nature (non-oval cross-section), their relationship to the LA/LV/aorta unit, and the course of the muscular fibers may all play a role.

The question may be raised as to how the results of the research can be applied in practice, what their clinical implications are, and in what way they can theoretically influence patient care. In our opinion, it is important to understand the relationships between the heart chambers and valves that make up the heart, like the MVA/AVA in healthy individuals, since knowing them allows us to understand whether there has been a change in the physiologic state in the presence of certain pathological conditions. Theoretically, these fats could facilitate the detection and understanding of observed abnormalities. Moreover, in real clinical practice, there is now a legitimate need to be able to detect such associations in the same individuals using non-invasive, reproducible imaging methods. This fact was confirmed in the present study, demonstrating that MVA and AVA can be examined simultaneously during 3DSTE. The findings also raise the possibility that simultaneous examination of other volumetric and functional parameters of heart chambers and valvular dimensions is possible, as demonstrated in previous results from the MAGYAR-Healthy Study [19].

It would be interesting to see the presented associations between the valvular annuli in certain pathologies, such as aortic or mitral incompetence or stenosis. The results presented suggest the importance of further investigations into this topic. Whether there are gender-related differences may also be an interesting question, as this was not analyzed in the present study due to the relatively low number of cases. Another question may be what results can be obtained with preserved or reduced LV-EF in the presence of heart failure. It would also be interesting to perform these measurements using other imaging techniques, such as magnetic resonance imaging with better resolution, in which the 3D shape of the valves (like the saddle-shape of the MVA) is also considered in the measurements.

**Limitation section.** The most important clinical and technical limitations are listed here:

- One of the most important technical limitations is the poorer image quality observed during 3DSTE as compared to that of 2D echocardiography, which still exists today. This may be due to the larger probe, stitching artifacts that may occur during the merging of digitally acquired 3D echocardiographic subvolumes, or the presence of arrhythmias, among other factors. Impact of sample rates and potential phase shifts on accurately capturing true maximum and minimum phases could significantly affect findings, which could be considered as the most important limitation of the present study [7,8,9,10,11].
- The present study did not aim to validate 3DSTE-derived measurement of MVA and AVA dimensions [12,13].
- Moreover, chamber quantification of any atria and ventricles was not aimed to be performed either, due to the fact that the present study focused solely on the simultaneous measurement of valvular annuli.
- The MVA has a spatial 3D saddle-like hyperbolic paraboloid shape, which was not taken into account in the present study; only its 2D projected dimensions were measured, which could have distorted the results [1,2,3,4].
- Valvular regurgitations were excluded only by visual assessment, and more advanced methods were not applied during assessments.
- Due to the use of certain statistical methods, the results obtained can only be interpreted in light of them. First, given the large number of comparisons, the risk of a type I error (false positives) in this study was very high. Second, forced stratification of continuous variables results in substantial information loss and reduces statistical power.

## 5. Conclusions

In healthy adults, an increase in any single parameter (diameter, area, or perimeter) of the AVA/MVA is significantly associated with an increase in all other parameters of that same annulus. This consistency holds true whether measured at end-diastole or end-systole. The study demonstrates a tendential increase in the dimensions of the MVA when AVA dimensions increase, and vice versa. This relationship is maintained regardless of which valve is larger or which cardiac phase is analyzed. However, in some special cases, these general rules were not obvious. The significant associationss reinforce the physiological concept that the MVA and AVA function as a coupled morphological entity rather than isolated structures. Moreover, all these parameters can be measured at the same time using the same 3D echocardiographic dataset during a detailed 3DSTE analysis.

## Figures and Tables

**Table 1:** Clinical and two-dimensional echocardiographic data.

Data	Measures
Clinical data	
n	134
Mean age (years)	31.0 (16.0)
Males (%)	73 (54)
Systolic blood pressure (mmHg)	116 ± 7
Diastolic blood pressure (mmHg)	76 ± 8
Heart rate (bpm)	72 ± 2
Weight (kg)	72.0 (19.0)
Height (cm)	172.0 (14.3)
Body mass index (kg/m^2^)	23.7 (4.3)
Two-dimensional echocardiographic data	
LA diameter (mm)	37.3 ± 3.6
LV end-diastolic diameter (mm)	48.4 ± 3.7
LV end-systolic diameter (mm)	32.1 ± 3.1
LV end-diastolic volume (mL)	107.4 ± 23.7
LV end-systolic volume (mL)	37.9 ± 9.0
Interventricular septum (mm)	9.2 ± 1.2
LV posterior wall (mm)	9.4 ± 1.5
LV ejection fraction (%)	65.0 ± 3.9
Early diastolic mitral inflow velocity—E (cm/s)	79.4 (22.0)
Late diastolic mitral inflow velocity—A (cm/s)	56.7 (15.2)

**Abbreviations:** LA = left atrial, and LV = left ventricular.

**Table 7:** Correlations between mitral valve and mitral/aortic valve dimensions respecting the cardiac cycle in healthy adults.

	MVA-D-S	MVA-A-S	MVA-P-S	MVA-D-D	MVA-A-D	MVA-P-D
**MVA-D-S**	1	**0.787** **(*p* < 0.001)**	**0.652** **(*p* < 0.001)**	**0.328** **(*p* < 0.001)**	**0322** **(*p* < 0.001)**	**0.274** **(*p* = 0.001)**
**MVA-A-S**	**0.787** **(*p* < 0.001)**	**1**	**0.956** **(*p* < 0.001)**	**0.449** **(*p* < 0.001)**	**0.511** **(*p* < 0.001)**	**0.480** **(*p* < 0.001)**
**MVA-P-S**	**0.652** **(*p* < 0.001)**	**0.956** **(*p* < 0.001)**	**1**	**0.442** **(*p* < 0.001)**	**0.528** **(*p* < 0.001)**	**0511** **(*p* < 0.001)**
**MVA-D-D**	**0.328** **(*p* < 0.001)**	**0.449** **(*p* < 0.001)**	**0.442** **(*p* < 0.001)**	1	**0.805** **(*p* < 0.001)**	**0.701** **(*p* < 0.001)**
**MVA-A-D**	**0.322** **(*p* < 0.001)**	**0.511** **(*p* < 0.001)**	**0.528** **(*p* < 0.001)**	**0.805** **(*p* < 0.001)**	1	**0.967** **(*p* < 0.001)**
**MVA-P-D**	**0.274** **(*p* = 0.001)**	**0.480** **(*p* < 0.001)**	**0.511** **(*p* < 0.001)**	**0.701** **(*p* < 0.001)**	**0.967** **(*p* < 0.001)**	1
**AVA-D-S**	−0.069 (*p* = 0.429)	0.087 (*p* = 0.317)	0.115 (*p* = 0.184)	0.089 (*p* = 0.308)	0.057 (*p* = 0.510)	0.041 (*p* = 0.642)
**AVA-A-S**	−0.052 (*p* = 0.552)	0.081 (*p* = 0.351)	0.102 (*p* = 0.241)	0.112 (*p* = 0.198)	0.096 (*p* = 0.269)	0.087 (*p* = 0.316)
**AVA-P-S**	−0.056 (*p* = 0.518)	0.104 (*p* = 0.231)	0.125 (*p* = 0.149)	0.106 (*p* = 0.223)	0.115 (*p* = 0.184)	0.112 (*p* = 0.197)
**AVA-D-D**	−0.057 (*p* = 0.515)	0.124 (*p* = 0.154)	0.137 (*p* = 0.114)	**0.180** **(*p* = 0.036)**	**0.188** **(*p* = 0.030)**	**0.186** **(*p* = 0.032)**
**AVA-A-D**	−0.062 (*p* = 0.479)	0.140 (*p* = 0.107)	0.166 (*p* = 0.055)	0.153 (*p* = 0.078)	0.136 (*p* = 0.118)	0.136 (*p* = 0.118)
**AVA-P-D**	−0.038 (*p* = 0.662)	0.168 (*p* = 0.052)	**0.194** **(*p* = 0.025)**	**0.202** **(*p* = 0.019)**	**0.184** **(*p* = 0.033)**	**0.187** **(*p* = 0.030)**

**Abbreviations.** MVA-D = mitral valve annular diameter, MVA-A = mitral valve annular area, MVA-P = mitral valve annular perimeter, AVA-D = aortic valve annular diameter, AVA-A = aortic valve annular area, AVA-P = aortic valve annular perimeter, S = end-systolic, and D = end-diastolic. Significance is represented by bold letters.

**Table 8:** Correlations between aortic valve dimensions and mitral/aortic valve dimensions with respect to the cardiac cycle in healthy adults.

	AVA-D-S	AVA-A-S	AVA-P-S	AVA-D-D	AVA-A-D	AVA-P-D
**MVA-D-S**	−0.069 (*p* = 0.429)	−0.052 (*p* = 0.552)	−0.056 (*p* = 0.518)	−0.057 (*p* = 0.514)	−0.062 (*p* = 0.479)	−0.038 (*p* = 0.662)
**MVA-A-S**	0.087 (*p* = 0.317)	0.081 (*p* = 0.351)	0.104 (*p* = 0.231)	0.124 (*p* = 0.154)	0.140 (*p* = 0.107)	0.168 (*p* = 0.052)
**MVA-P-S**	0.115 (*p* = 0.184)	0.102 (*p* = 0.241)	0.125 (*p* = 0.149)	0.137 (*p* = 0.114)	0.166 (*p* = 0.055)	**0.194** **(*p* = 0.025)**
**MVA-D-D**	0.089 (*p* = 0.308)	0.112 (*p* = 0.198)	0.106 (*p* = 0.223)	**0.180** **(*p* = 0.038)**	0.153 (*p* = 0.078)	**0.202** **(*p* = 0.019)**
**MVA-A-D**	0.057 (*p* = 0.510)	0.096 (*p* = 0.269)	0.115 (*p* = 0.184)	**0.188** **(*p* = 0.030)**	0.136 (*p* = 0.118)	**0.184** **(*p* = 0.033)**
**MVA-P-D**	0.041 (*p* = 0.642)	0.087 (*p* = 0.316)	0.112 (*p* = 0.197)	**0.186** **(*p* = 0.032)**	0.136 (*p* = 0.118)	**0.187** **(*p* = 0.030)**
**AVA-D-S**	**1**	**0.866** **(*p* < 0.001)**	**0.878** **(*p* < 0.001)**	**0.636** **(*p* < 0.001)**	**0.685** **(*p* < 0.001)**	**0.603** **(*p* < 0.001)**
**AVA-A-S**	**0.866** **(*p* < 0.001)**	**1**	**0.986** **(*p* < 0.001)**	**0.696** **(*p* < 0.001)**	**0.777** **(*p* < 0.001)**	**0.738** **(*p* < 0.001)**
**AVA-P-S**	**0.878** **(*p* < 0.001)**	**0.986** **(*p* < 0.001)**	**1**	**0.704** **(*p* < 0.001)**	**0.792** **(*p* < 0.001)**	**0.741** **(*p* < 0.001)**
**AVA-D-D**	**0.636** **(*p* < 0.001)**	**0.696** **(*p* < 0.001)**	**0.704** **(*p* < 0.001)**	**1**	**0.836** **(*p* < 0.001)**	**0.811** **(*p* < 0.001)**
**AVA-A-D**	**0.685** **(*p* < 0.001)**	**0.777** **(*p* < 0.001)**	**0.792** **(*p* < 0.001)**	**0.836** **(*p* < 0.001)**	**1**	**0.785** **(*p* < 0.001)**
**AVA-P-D**	**0.603** **(*p* < 0.001)**	**0.738** **(*p* < 0.001)**	**0.741** **(*p* < 0.001)**	**0.811** **(*p* < 0.001)**	**0.785** **(*p* < 0.001)**	**1**

**Abbreviations.** MVA-D = mitral valve annular diameter, MVA-A = mitral valve annular area, MVA-P = mitral valve annular perimeter, AVA-D = aortic valve annular diameter, AVA-A = aortic valve annular area, AVA-P = aortic valve annular perimeter, S = end-systolic, and D = end-diastolic. Significance is represented by bold letters.

**Table 9:** Intra- and interobserver variability for three-dimensional speckle-tracking echocardiography-derived mitral and aortic valve annular dimensions.

	Intraobserver Agreement		Interobserver Agreement	
	Mean ± 2SD Difference in Values Obtained by 2 Measurements of the Same Observer	ICC Between Measurements of the Same Observer	Mean ± 2SD Difference in Values Obtained by 2 Observers	ICC Between Independent Measurements of 2 Observers
MVA-D-S (cm)	−0.03 ± 0.12 cm	0.95 (*p* < 0.0001)	0.04 ± 0.15 cm	0.97 (*p* < 0.0001)
MVA-A-S (cm^2^)	−0.03 ± 0.20 cm^2^	0.97 (*p* < 0.0001)	−0.04 ± 0.59 cm^2^	0.96 (*p* < 0.0001)
MVA-P-S (cm)	0.05 ± 0.79 cm	0.97 (*p* < 0.0001)	0.05 ± 0.51 cm	0.96 (*p* < 0.0001)
MVA-D-D (cm)	0.02 ± 0.16 cm	0.96 (*p* < 0.0001)	0.03 ± 0.18 cm	0.98 (*p* < 0.0001)
MVA-A-D (cm^2^)	−0.03 ± 0.83 cm^2^	0.96 (*p* < 0.0001)	0.04 ± 0.58 cm^2^	0.96 (*p* < 0.0001)
MVA-P-D (cm)	−0.03 ± 0.80 cm	0.97 (*p* < 0.0001)	−0.08 ± 0.68 cm	0.96 (*p* < 0.0001)
AVA-D-S (cm)	0.03 ± 0.30	0.91 (*p* < 0.0001)	0.04 ± 0.28	0.94 (*p* < 0.0001)
AVA-A-S (cm^2^)	0.09 ± 0.72	0.91 (*p* < 0.0001)	0.10 ± 0.74	0.93 (*p* < 0.0001)
AVA-P-S (cm)	−0.03 ± 0.58	0.91 (*p* < 0.0001)	0.02 ± 0.49	0.93 (*p* < 0.0001)
AVA-D-D (cm)	−0.03 ± 0.23	0.89 (*p* < 0.0001)	−0.06 ± 0.18	0.90 (*p* < 0.0001)
AVA-A-D (cm^2^)	−0.10 ± 0.58	0.93 (*p* < 0.0001)	−0.11 ± 0.50	0.93 (*p* < 0.0001)
AVA-P-D (cm)	−0.07 ± 0.59	0.92 (*p* < 0.0001)	−0.11 ± 0.62	0.94 (*p* < 0.0001)

**Abbreviations.** ICC = interclass correlation coefficient, MVA-D = mitral valve annular diameter, MVA-A = mitral valve annular area, MVA-P = mitral valve annular perimeter, AVA-D = aortic valve annular diameter, AVA-A = aortic valve annular area, AVA-P = aortic valve annular perimeter, S = end-systolic, and D = end-diastolic.

## Data Availability

The data presented in this study are available on request from the corresponding author.
